# Supplementary material for: Study protocol for the ‘preventing functional decline in acutely hospitalised older patients (PREV_FUNC)’ study: effects of two multicomponent exercise programmes on physical function – a three-armed randomised controlled trial
Source: BMJ Open. 2023 Aug 22;13(8):e070885. doi: 10.1136/bmjopen-2022-070885 (PMC10445339; doi:10.1136/bmjopen-2022-070885)
Supplement: Supplementary data [file bmjopen-2022-070885supp001.pdf]

The "Preventing functional decline in acutely hospitalized older patients (PREV_FUNC)" study

Information about participation in research project

We would like to ask you if you would like to participate in a research project. In this document you will receive information about the project and what it means to participate.

What kind of project is it and why do you want me to participate?

Physical activity is important for good health. According to research, physical exercise during hospitalization can be effective in improving independence and function. The aim is therefore to explore the effect on physical function of two different exercise interventions during and after hospital stay compared to usual care. To participate, you must be 75 years of age or older and meet set criteria for participating in the study. You are asked to participate since you meet the criteria. If you accept participation in the study, you will either receive usual care or one of the two exercise interventions. One exercise intervention contains strength, balance and walking exercises and the other exercise intervention contains chair-stand exercises and walking. The exercise you receive through your participation in the project is in addition to the usual care. Whether you receive usual care, or one of the two training interventions is determined by the period of time you are hospitalized.
The research principal for the project is Karolinska Institutet. Research principal means the organization responsible for the study. The application has been approved by the Ethics Review Authority, the diary number for the review at the Ethics Review Authority is 2022-03032-01.

How is the study conducted?

Your participation in this research study will mean that at the beginning and end of your hospital stay and approximately three months after discharge, you will be asked to do tests and answer questionnaires. The tests aim to examine walking speed, balance, leg strength, grip strength, arm and calf circumference, as well as ability to perform activities of daily living. The questionnaires aim to ask you how you perceive your health and quality of life in various areas. This examination will take approximately 30 minutes to complete. The examination, which is carried out three months after discharge, can be done in your home or at the hospital as agreed. Other information such as age, sex, your medical condition, length of hospital stay, and place of discharge will be collected from your medical record. If you choose to participate, you also agree to us collecting information about your visits and diagnoses from the healthcare system up to one year after you have been discharged from the hospital.
The individualized exercise program involves exercising for 40 minutes divided into two sessions per day. Exercises that can be included are strength, balance, and walking. The second exercise program involves exercising for 20–30 minutes spread over four sessions per day. This exercise includes getting up from a chair and walking. You will receive a simple self-training program with these exercises that you continue with at home for six weeks. You register the exercise in a logbook with a cross. All exercise at the hospital is adapted to your ability. The training is led and documented by certified personnel.

Possible consequences and risks of participating in the study

Tests of balance can challenge your balance. In order not to risk losing your balance and fall, there will be a person close to you who will be ready to assist you.

The “Preventing functional decline in acutely hospitalized older patients (PREV_FUNC)” study

What will happen to my data?
The information collected about you in the study will be protected by confidentiality according to the Swedish Publicity and Confidentiality Act, which means that no unauthorized persons may access the information. The collected material will be coded, and the data and code key will be kept separately locked according to standard practice. This means that no one except the responsible researchers can identify you as a person if necessary. Everyone who works on the study is bound by confidentiality. All results from the study will be presented at group level. If during the examination we discover something that needs to be followed up, we can arrange contact with the relevant care facility.
Responsible for your personal data is Karolinska Institutet as the research principal. According to the EU's data protection regulation, you have the right to access the information about you, that is handled in the study, free of charge, and if necessary to have any errors corrected. You can also request that information about you be deleted and that the processing of your personal data be restricted. If you want to access the data, please contact Anna-Karin Welmer, Karolinska Institutet, Department of Physiotherapy, Alfred Nobels allé 23, 141 83 Huddinge, Sweden, telephone number: +46 8 524 888 04. The code key will be destroyed after 10 years. Thereafter, it is not possible to hand out any register extract. The information you provide is protected according to chapter 24. paragraph 8, of the Swedish Publicity and Privacy Act (2009:400) and the EU's data protection regulation, GDPR. The Data Protection Officer can be reached at dataskyddsombud@ki.se. If you are dissatisfied with the way your personal data is processed, you have the right to file a complaint with the Swedish Authority for Privacy Protection (IMY), which is the supervisory authority.

How do I get information about the results of the study?
The study results will be reported at group level and published in scientific journals, popular science literature and at conferences nationally and internationally. Your identity will not be traceable in these reports. If you as a research participant want to get your results out, please contact the researcher responsible for the project.

Insurance and compensation
Compensation is not eligible. Patient insurance covers your participation in the study.

Participation is voluntary
Your participation is voluntary, and you can choose to cancel your participation at any time. If you choose not to participate or wish to cancel your participation, you do not need to state why, and it will not affect your future care or treatment. If you wish to cancel your participation, please contact the responsible researchers (see below).

Responsible for the study
For further information, please contact:

Anna-Karin Welmer<br>Senior Lecturer, Associate Professor, Physiotherapist<br>anna-karin.welmer@ki.se<br>+46 8 524 888 04	Anne-Marie Boström<br>Senior Lecturer, Associate Professor, RN<br>anne-marie.bostrom@ki.se<br>+46 760 519513	Linda Sandberg<br>PhD, Occupational Therapist<br>linda.m.sandberg@ki.se

The "Preventing functional decline in acutely hospitalized older patients (PREV_FUNC)" study

Consent to participate in the study

I have received oral and written information about the study and have had the opportunity to ask questions. I get to keep the written information.

☐ I agree to participate in the study *Preventing functional decline in acutely hospitalized older patients (PREV_FUNC)*

☐ I agree to data about me being collected and processed in the manner described in the research participant information

Place and date	Signature
	Name claification
