# Supplementary material for: Study protocol for the ‘preventing functional decline in acutely hospitalised older patients (PREV_FUNC)’ study: effects of two multicomponent exercise programmes on physical function – a three-armed randomised controlled trial
Source: BMJ Open. 2023 Aug 22;13(8):e070885. doi: 10.1136/bmjopen-2022-070885 (PMC10445339; doi:10.1136/bmjopen-2022-070885)
Supplement: Author's manuscript [file bmjopen-2022-070885.draft_revisions.pdf]

BMJ Open is committed to open peer review. As part of this commitment we make the peer review history of every article we publish publicly available.

When an article is published we post the peer reviewers' comments and the authors' responses online. We also post the versions of the paper that were used during peer review. These are the versions that the peer review comments apply to.

The versions of the paper that follow are the versions that were submitted during the peer review process. They are not the versions of record or the final published versions. They should not be cited or distributed as the published version of this manuscript.

BMJ Open is an open access journal and the full, final, typeset and author-corrected version of record of the manuscript is available on our site with no access controls, subscription charges or pay-per-view fees (http://bmjopen.bmj.com).

If you have any questions on BMJ Open's open peer review process please email
info.bmjopen@bmj.com

BMJ Open

A study protocol for the "Preventing functional decline in acutely hospitalized older patients (PREV_FUNC)" study – Effects of two multicomponent exercise programs on physical function: a three-armed randomized controlled trial

Journal:	*BMJ Open*
Manuscript ID	bmjopen-2022-070885
Article Type:	Protocol
Date Submitted by the Author:	08-Dec-2022
Complete List of Authors:	Welmer, Anna-Karin; Karolinska Institutet, Department of Neurobiology, Care Sciences and Society<br>Sandberg, Linda; Karolinska Institutet, Department of Neurobiology, Care Sciences and Society<br>Sandlund, Christina; Karolinska Institutet, Department of Neurobiology, Care Sciences and Society<br>Björck, Caroline; Uppsala University, Department of Women´s and Children´s Health<br>Hagströmer, Maria; Karolinska Institutet, Department of Neurobiology Care Sciences and<br>Hamilton, Julia; Sabbatsberg Hospital, Department of Geriatric Medicine<br>Helgstrand, Gunilla; Sabbatsberg Hospital, Department of Geriatric Medicine; Karolinska Institutet, Department of Neurobiology, Care Sciences and Society<br>Lindgren , Charlotte; Dalengeriatriken Aleris Närsjukvård AB, Department of Geriatric medicine<br>Nordstrand, Linda; Karolinska University Hospital; Karolinska Institutet, Department of Neurobiology, Care Sciences and Society<br>Sandstedt, Petter; Karolinska University Hospital<br>Kivipelto, Miia; Karolinska Institutet, Division of Geriatric Epidemiology<br>Boström, Anne-Marie; Karolinska Institutet, Department of Neurobiology, Care Sciences and Society; Karolinska University Hospital, Theme Inflammation and Aging
Keywords:	GERIATRIC MEDICINE, PREVENTIVE MEDICINE, Clinical trials < THERAPEUTICS

SCHOLARONE™
Manuscripts

A study protocol for the "Preventing functional decline in acutely hospitalized older patients (PREV_FUNC)" study – Effects of two multicomponent exercise programs on physical function: a three-armed randomized controlled trial

Anna-Karin Welmer[1,2,3], Linda Sandberg[1,4], Christina Sandlund[1,5,6], Caroline Björck[7,8], Maria Hagströmer[1,9], Julia Hamilton[10], Gunilla Helgstrand[10,11], Charlotte Lindgren[4], Linda Nordstrand[3,11], Petter Sandstedt[3], Miia Kivipelto[12,13,14], Anne-Marie Boström[13,14,15]

[1] Division of Physiotherapy, Department of Neurobiology, Care Sciences and Society, Karolinska Institutet, Stockholm, Sweden

[2] Aging Research Center, Department of Neurobiology, Care Sciences and Society, Karolinska Institutet and Stockholm University, Stockholm, Sweden

[3] Women´s Health and Allied Health Professionals Theme, Medical Unit Medical Psychology, Karolinska University Hospital, Stockholm, Sweden

[4] Department of Geriatric medicine, Dalengeriatriken Aleris Närsjukvård AB, Stockholm, Sweden

[5] Division of Family Medicine and Primary Care, Department of Neurobiology, Care Sciences and Society, Karolinska Institutet, Stockholm, Sweden

[6] Academic Primary Health Care Centre, Region Stockholm, Stockholm, Sweden

[7] Department of Women´s and Children´s Health, Uppsala University, Uppsala, Sweden

[8] Centre for Research and Development, County Council of Gävleborg, Gävle, Sweden

[9] Academic Primary Health Care Centre, Region Stockholm, Stockholm, Sweden

[10] Department of Geriatric Medicine, Sabbatsberg Hospital, Region Stockholm, Stockholm, Sweden

For peer review only - http://bmjopen.bmj.com/site/about/guidelines.xhtml

[11] Division of Occupational Therapy, Department of Neurobiology, Care Sciences and Society, Karolinska Institutet, Stockholm, Sweden

[12] Division of Clinical Geriatrics, Department of Neurobiology, Care Sciences and Society, Karolinska Institutet, Stockholm, Sweden

[13] Theme Inflammation and Aging, Medical Unit Aging, Karolinska University Hospital, Huddinge, Sweden

[14] Research and Development Unit, Stockholm´s Sjukhem, Stockholm, Sweden

[15] Division of Nursing, Department of Neurobiology, Care Science and Society, Karolinska Institutet, Huddinge, Sweden

Correspondence to:

Anna-Karin Welmer, PhD, Associate Professor

Karolinska Institutet

Department of Neurobiology, Care Sciences and Society

Division of Physiotherapy 23100

SE-141 83 Huddinge, Sweden

Email: anna-karin.welmer@ki.se

Phone: +46 8 524 888 04

For peer review only

Abstract

Introduction: Acutely hospitalized older patients are often frail and at an increased risk of declining physical function. Previous studies suggest that exercise might mitigate the risk of physical impairment; however, further research is needed to compare the effectiveness of different types of exercise interventions. In this paper we report a protocol for a study that aims to examine 1) if multicomponent exercise interventions (including both mobility and strengthening exercises) have effects on physical function compared to usual care in older adults, and 2) if a comprehensive multicomponent exercise program is more effective than a simple multicomponent exercise program including only walking and rising from a chair.

Methods and analysis: This is a three-armed randomized controlled trial, with two intervention groups (comprehensive and simple exercise program) and a control group receiving usual care. We will include 320 participants aged ≥75 years from geriatric medical wards of four hospitals in Stockholm, Sweden. Assessments will be conducted at hospital admission, discharge and three months thereafter concerning physical function (primary outcome), activities of daily living, health-related quality of life, sarcopenia, and falls. Number of re-admissions will be registered up to 1 year after discharge. Data will be analyzed with linear mixed effects models, according to the intention-to-treat approach.

Ethics and dissemination: Ethical approval for this study has been granted by the Swedish Ethical Review Authority (approval number 2022-03032-01). Data collection will consider the information requirement, the requirement of consent, confidentiality obligations, and the utilization requirement. Study findings will be disseminated through multiple channels, including scientific publications and conferences, and workshops with healthcare professionals and the public.

Trial registration: NCT05366075, ClinicalTrials.gov, 4 May 2022, https://clinicaltrials.gov/

For peer review only - http://bmjopen.bmj.com/site/about/guidelines.xhtml

Strengths and limitations of this study

• Study participants will be consecutively recruited at several hospitals, so that the sample will be representative of older hospitalized patients

• The project has a clear interdisciplinary and practice-oriented focus, which will facilitate implementation

• Both exercise interventions use none or low technology exercise equipment that is already available in most hospital settings or can be purchased for a low price

• The block design may introduce selection bias in the study; however, an advantage is that it avoids co-presence of participants from both groups at the same department

• Due to the difficulties of double-blinding, a single-blind study design is used, in which research staff blinded to group allocation will conduct the assessments

Keywords: Exercise, Hospitalization, Older Adults, Physical Functional Performance, Randomized Controlled Trial

Word count: 2767 words

For peer review only

For peer review only - http://bmjopen.bmj.com/site/about/guidelines.xhtml

Introduction

Hospitalization because of acute medical illness is associated with several negative health consequences in older adults, even if the illness that caused the admission is successfully treated. Approximately, half of adults aged 65 years and older show a decline in physical function following acute hospitalization (1). Many patients do not regain pre-hospitalization function, lose independence, and have high risk of reduced well-being, readmissions, and death after discharge (2, 3).

Acutely hospitalized older patients often have frailty, a reduced resilience and an increased vulnerability to stressors, and several concurrent diseases (3). Furthermore, acutely hospitalized older people spend most of their hospital time in bed, even if they can walk independently (4). Such short periods of physical inactivity may exacerbate the age-related loss of muscle mass, strength, and physical function (5, 6). It has been suggested that relatively small amounts of exercise might mitigate these deficits (7, 8), emphasizing the importance of identifying easy-to-use exercise interventions for older hospitalized people.

A recent meta-analysis suggested that in-hospital exercise interventions are effective for the improvement of functional independence; however, not all types of exercise interventions seem to provide the same effect (9). In a recent randomized controlled trial (RCT), Martinez-Velilla, et al. demonstrated that a comprehensive exercise program, consisting of several exercises including supervised progressive resistance, balance, and walking training, provided significant health benefits over usual care in acutely hospitalized patients aged 75 years and older (10). Also, in another recent RCT, Ortiz-Alonso, et al. showed that a relatively simple multicomponent intervention solely consisting of walking and rising from a chair decreased the risk of dependence in activities of daily living (ADL) in acutely hospitalized patients older than 75 years (11). So far, it is not known whether such a simple exercise program could yield similar health benefits as a more comprehensive intervention, such as the one by Martinez-

For peer review only - http://bmjopen.bmj.com/site/about/guidelines.xhtml

Velilla, et al. Identifying the most efficient interventions to reduce health consequences of acute hospitalization in older adults should be a high priority in society, especially considering the increasing older population.

In this paper we present a study protocol for the "Preventing functional decline in acutely hospitalized older patients (PREV_FUNC)" study – a three-armed randomized controlled trial that evaluates the effects of two multicomponent exercise programs on physical function.

For

Methods and analysis

Objectives

The main aims of the PREV_FUNC study are to 1) evaluate the effect of multicomponent exercise interventions (including both mobility and strengthening exercises) on physical function (primary outcome), ADL, health-related quality of life (HRQL), sarcopenia, falls, readmissions, and mortality in comparison with usual care in acutely hospitalized people aged 75 years and older, and 2) if a comprehensive multicomponent exercise program including several exercises is more effective than a simple multicomponent exercise program including only walking and sit-to-stand exercises. An initial pilot of the PREV_FUNC study suggested that the intervention was feasible, safe, and acceptable to the participants (unpublished data).

Trial design

The PREV_FUNC study will be conducted in accordance with the Consolidated Standards of Reporting Trials (12). It is a three-arm single blind block randomized multi-center controlled trial. Participants will be included consecutively from geriatric medical wards of four hospitals in Stockholm, Sweden. A flowchart of the study participants is shown in Figure 1. The patients will be included in the control group or one of the intervention groups in a time-dependent manner: participants will be recruited to each group during 2-3-week blocks, that will be repeated until the end of the study. The order of the groups will be randomized for

For peer review only - http://bmjopen.bmj.com/site/about/guidelines.xhtml

each study site (randomization will be done by the responsible researchers). The block design has been chosen to avoid co-presence of participants from both groups at the same department; thus, to prevent that the exercises in the intervention groups will spread to treatment as usual and risk to contaminate the control group. Eligible subjects will be invited to participate in the study within 36 hours of admission and the baseline examination will be conducted directly after the inclusion. The study has been registered at clinicaltrials.gov. This protocol is reported in accordance with the Standard Protocol Items: Recommendations for Interventional Trials.

Inclusion and exclusion criteria

Inclusion criteria for participation are age 75 years and older, ability to stand up from a sitting position independently or with minimal personal help and to communicate and collaborate with the research staff (e.g., patients with severe confusion will be excluded). We will exclude patients with terminal illness or any major medical condition that contraindicates exercise, those living in nursing home, or those previously included in the study.

Interventions

The interventions will start the same day as the baseline examination or the day after and will be performed on all weekdays until discharge. Intervention 1 is adapted from the exercise program by Ortiz-Alonso, et al. (11), and Intervention 2 is adapted from the exercise program by Martinez-Velilla, et al. (9). The interventions will be offered in addition to usual care.

Intervention 1 (simple exercise program) (11). The intervention will include up to four sessions per day (total duration 20-30 minutes/day). This intervention consists of sit-to stand exercises and walking along the corridor of the ward (for those who can walk). The exercises will mainly be led by physiotherapists, and, when suitable, it will be performed in connection with regular routines in the usual care (Table 1). At discharge, the participants will be encouraged to continue with the exercise program at home, three times per week during six

For peer review only - http://bmjopen.bmj.com/site/about/guidelines.xhtml

weeks. In addition to oral instructions, they will receive written instructions with pictures on how to perform the training.

Intervention 2 (comprehensive exercise program) (9). This intervention consists of two daily sessions (morning and afternoon) of 20 minutes' duration each. The morning session includes individualized supervised progressive resistance, balance, and walking training exercises, tailored to each participant's capacity. The resistance training includes using weight cuffs, involving mainly lower-extremity muscles. Balance and gait exercises includes different exercises such as line walking, stepping practice, and walking with small obstacles. The evening session consists of functional exercises using light loads, such as knee extension and flexion, hand training with a ball, and daily walking exercises (for those who can walk). The exercises will be led by physiotherapists (Table 1).

Control group. The control group will receive usual care, which may include physical rehabilitation when needed, such as gait retraining. We expect that the participants often have a limited amount of physical activity during the hospital stay (13).

Daily logbooks will be used during the hospital stay to register the number of attended exercise sessions for the intervention groups as well as treatment safety for all groups (by registering adverse events during the hospital stay, e.g., falls). Furthermore, training diaries will be used during six weeks to register the self-training conducted at home in intervention 1.

For peer review only

For peer review only - http://bmjopen.bmj.com/site/about/guidelines.xhtml

Table 1. Overview of the interventions.

Intervention	Timing/frequency and duration of exercises	Description	Dose
**Intervention 1**<br>(Simple exercise program)	Up to 4 times/day<br>(Total duration 20-30 minutes/day)	• **Sit-to-stand exercises.** Rising from a chair and sitting down again repeatedly, using armrest and assistance if needed<br>• **Walking.** Walking along the corridor, using walking aid and assistance if needed	1-3 sets of up to 10 repetitions, depending on the participant's ability<br>3-10 min per session, with resting periods if needed
**Intervention 2**<br>(Comprehensive exercise program)	Before lunch<br>(Duration 20 minutes)	*Strengthening exercises*<br>• **Sit-to-stand exercises.** Rising from a chair and sitting down again repeatedly, using armrest and assistance if needed<br>• **Knee extensions.** Knee extensions in a sitting position, with a weight cuff on the ancle. Repeat with both legs<br>• **Standing push-ups.** Bending and stretching arms leaning on a wall	1-3 sets of up to 10 repetitions, depending on the participant's ability
		Balance exercises<br>• **Semi-tandem stance.** One foot placed in front of the other foot, with the big toe of the back foot in the groove of the front foot. Repeat on the other side<br>• **Line walking.** Walking in a straight line, placing one foot in front of the other, and vice versa<br>• **Walking with small obstacles**. Walking around small obstacles and/or stepping over small obstacles<br>• **Stepping practice**. Stepping up and down on a step board or staircase<br>• **Weight transfer.** Standing up, transferring the weight from one leg to the other<br>• **Proprioceptive exercises**. Walking on an unstable surface	Dose adapted to the participant's ability and time frame
	Afternoon	• **Knee extensions.** Knee extensions in a sitting position. Repeat with both legs<br>• **Knee flexion.** Standing knee flexions, holding on to a chair. Repeat with both legs	1-3 sets of up to 10 repetitions for, depending on the participant's ability

For peer review only - http://bmjopen.bmj.com/site/about/guidelines.xhtml

	(Duration 20 minutes)	• **Hip abduction.** Standing hip abduction with the participant standing, holding on to a chair. Repeat with both legs<br>• **Hand training** with handgrip ball<br>• **Walking.** Walking along the corridor, using walking aid and assistance if needed	

For peer review only

Data collection

Physical function, ADL, HRQL, sarcopenia and falls will be assessed at baseline (hospital admission), discharge, and three months after discharge. Research staff blinded to group allocation will conduct the tests and administer the questionnaires at the hospital (for the baseline and discharge evaluations) and at the participants´ homes (for the three-month evaluation). The six-month and one-year evaluations will be conducted using patient records (for hospital readmissions and mortality) (Table 2).

The primary outcome will be lower extremity physical function, assessed according to the Short Physical Performance Battery (SPPB) (14). The SPPB has three components: standing balance, walking speed, and the chair stand test for lower extremity strength. It has been validated in frail older adults, is sensitive to change, and predicts a wide range of clinical outcomes (15, 16).

Secondary outcomes will be ADL (according to the Barthel Index) (17), HRQL (according to the EuroQol–5 Dimension) (18), presence of sarcopenia (defined by handgrip strength and calf circumference and arm circumference), according to the European Working Group's revised criteria on Sarcopenia in Older People (19), and number of falls during follow-up (according to the logbooks at the hospital and self-report at the three-month follow-up). Data on vital status and number of readmissions up to one year after discharge will be collected using patient records.

Baseline data will also be collected from patient records on demographic data (age, sex, and cohabitation status), reason for hospital admission, comorbidities, frailty status, and nutritional status. Frailty status will be defined according to the Clinical Frailty Scale (20) and nutritional status will be defined according to the Mini Nutritional Assessment-Short Form (21). Type and amount of nutritional supplementation consumed during the hospitalization (if applicable) will be recorded in a logbook. Falls in the previous year will be assessed

For peer review only - http://bmjopen.bmj.com/site/about/guidelines.xhtml

For peer review only

according to self-report at baseline. Length of hospital stay, and discharge destination will be registered at discharge.

Table 2. Time of assessment of the different variables in the study.

	Time point				
**Assessment**	Baseline (hospital admission)	After training/control period (discharge)	3 months after discharge	6 months after discharge	1 year after discharge
**Primary outcome**					
Physical function	X	X	X		
**Secondary outcomes**					
Activities of daily living	X	X	X		
Health-related quality of life	X	X	X		
Sarcopenia	X	X	X		
Falls after inclusion		X	X		
Hospital readmissions			X	X	X
Mortality			X	X	X
**Additional data**					
Frailty status	X				
Nutritional status	X				
Nutritional supplementation	Continuously collected during the hospitalization				
Falls in the previous year	X				
Demographic data	X				
Reason for hospital admission	X				
Comorbidities	X				
Length of hospital stay		X			
Discharge destination		X			

For peer review only - http://bmjopen.bmj.com/site/about/guidelines.xhtml

Data analysis

Power of the study. We estimate that 264 participants should be included in the main RCT to enable detection of a clinically meaningful difference of one point in the main outcome (SPPB) at three months after discharge (22), when power is 0.80 and alpha is 0.05 (three groups comparison). To further allow for approximately 20% deaths or dropouts over three months, 320 participants should be included in the study.

We will use the intention-to-treat approach. Between-group comparisons of change in the continuous variables from baseline to follow-up, will be conducted using linear mixed effects models, with adjustments for baseline scores. To account for deaths and loss to follow-up, we will model the group comparisons through joint models, which will be performed by simultaneously estimating both the linear mixed model and a Cox's proportional hazard regression model. Group differences in the number of readmissions will be examined using ordinal logistic regression models. Binary logistic regression models will be employed to examine between group differences in binary outcomes, and Cox's proportional hazard regression model will be used for mortality data. Multiplicative interactions between sex and group allocation in relation to the outcomes will also be examined. Statistical analyses will be performed with Stata and R.

Patient and public involvement

The project is conducted in close dialog between researchers, clinicians, and health care managers, of different disciplines. Thus, the project has a clear interdisciplinary and practice-oriented focus, which will facilitate its implementation. Interdisciplinary collaboration is a crucial part of the study since hospital care requires involvement of several professions to meet the needs of the older adults, who often have several concurrent health problems. Furthermore, we plan to conduct qualitative interviews on the participants' views of the interventions.

For peer review only - http://bmjopen.bmj.com/site/about/guidelines.xhtml

Trial status

This is version 1 of the study protocol. Recruitment for the study is ongoing and is planned between September 2022 and December 2023. Any deviations to this protocol will be updated on clinicaltrials.gov, and the changes will be reported when disseminating the results.

Ethics and dissemination

Ethical approval for this study has been granted by the Swedish Ethical Review Authority (approval number 2022-03032-01). Data collection will consider the information requirement, the requirement of consent, confidentiality obligations, and the utilization requirement. The participants will receive verbal and written information and informed written consent will be collected by researchers. All data collected is pseudonymized and stored in paper and digital format in accordance with the General Data Protection Regulation. Only the responsible researchers will have full access to the data. Study findings will be disseminated through multiple channels, including scientific publications and conferences, and workshops with healthcare professionals and the public. We will follow the Vancouver recommendations for authorship.

Discussion

The goal of this project is to provide knowledge on how to prevent physical impairment in acutely hospitalized patients aged 75 years and older. Although several studies have shown beneficial effects of community-based exercise programs in older adults, less attention have been given to exercise programs during hospitalization because of acute medical illness (23). To date, there is no consensus concerning the most effective exercise intervention to prevent functional decline in acutely hospitalized older adults (9).

For peer review only - http://bmjopen.bmj.com/site/about/guidelines.xhtml

Acutely hospitalized patients aged 75 years and older often suffer from frailty and multimorbidity and consume a large proportion of the resources of health care (24). Current guidelines for care of various diseases are mainly based on research performed on younger people without severe comorbidity and cannot always be generalized to older patients. This project may provide a novel strategy to maintain or improve physical function in this group of frail older adults. If the study will show positive effects, we plan to further analyze its cost-effectiveness in subsequent studies. If our results are positive, this might lead to a change in clinical practice where multicomponent exercises are incorporated in routine care. Our outcome, physical function, is an important component of healthy aging and a strong predictor of future health status and mortality following hospitalization in older adults (15, 16).

Our interventions are based on two previous studies conducted among Spanish hospitalized older adults (10, 11). The results from these studies indicate that multicomponent exercises are safe and effective in counteracting functional limitations in older hospitalized people. Research is however needed on the generalizability of these approaches to other settings. Furthermore, it is not known whether these approaches are equally efficient in counteracting functional limitations in older hospitalized people. In the present study, we have modified the intervention by Martinez-Velilla, et al. (10), such that the resistance training is done using weight cuffs instead of resistance training machines. This adaption was done to enhance implementation in clinical settings. Both exercise interventions examined in this study use none or low technology exercise equipment that is already available in most hospital settings or can be purchased for a low price. Furthermore, we modified the intervention by Ortiz-Alonso, et al. such that we increased the total duration of the training from 20 minutes per day to 20-30 minutes per day (11). This was done to make the two interventions more comparable in terms of daily exercise time.

For peer review only - http://bmjopen.bmj.com/site/about/guidelines.xhtml

This study has some limitations. First, due to the difficulties of double-blinding, a single-blind study design is used, in which researchers blinded to group allocation will conduct the tests and administer the questionnaires. Second instead of a classic randomization we have chosen a block design, which may introduce selection bias in the study. However, an advantage of the block design is that it avoids co-presence of participants from both groups at the same department; and thus, prevents that the exercises in the intervention groups will spread to treatment as usual and risk to contaminate the control group. Moreover, the study will include participants who are able to stand up from a sitting position and to communicate and collaborate with the research team, which could limit the generalizability to those with relatively higher levels of functioning. However, the study participants will be consecutively recruited at several hospitals, so that the sample will be representative of older hospitalized patients.

A possible challenge in this study may be the gradual change towards shortened length of hospital stay for geriatric inpatients in Sweden (25). A further shortened average length of hospital stay may limit the possibilities of showing significant effects of the interventions during the hospital stay. Thus, in intervention 1 the participants will be encouraged to continue with the exercises at home. In future studies, we plan to explore prerequisites for a transitional care intervention, in which physical exercise is performed during acute hospitalization and as self-training at home with support from primary care. This three-armed intervention is a first step towards finding the best strategies to maintain or improve physical function among in acutely hospitalized older adults.

In summary, the PREV_FUNC study has the potential to provide new knowledge on how to maintain and improve physical function in acutely hospitalized older patients. The number of hospital admissions are likely to increase as the population age. Identifying the most efficient

For peer review only - http://bmjopen.bmj.com/site/about/guidelines.xhtml

For peer review only

interventions to reduce health consequences of acute hospitalization in older adults is thus highly relevant from clinical and public health perspectives.

Author contributions

AKW, AMB and LS designed the protocol and methods of the study. AKW, LS, CS, CB, MH, JH, GH, CL, LN, PS, MK and AMB contributed with the writing the manuscript. All authors read and approved the final manuscript.

Funding statement

The PREV_FUNC study is supported by grants provided by the Swedish Research Council for Health, Working Life and Welfare, Grant Number 2021-01788; Region Stockholm (ALF project), Grant Number FoUI-960460, Center for Innovative Medicine (CIMED), Region Stockholm, Grant Number FoUI-961330; and Konung Gustaf V:s och Drottning Victorias Frimurarestiftelse (PI AKW). In addition, MK was supported by Stiftelsen Stockholms Sjukhem, CIMED and the Swedish Research Council, Grant Number 2017-0610. The funders have no role in design of the study, collection, management, analysis, interpretation of data, writing manuscripts, or in the decision to submit for publication.

Competing interests

The authors declare that they have no competing interests.

Data statement

Data sharing is not applicable to this article as no datasets were generated or analyzed during the current study. The data that will be collected will be available upon request, and requests for access to the data can be put to the Research Data Office (rdo@ki.se) at Karolinska Institutet and will be handled according to the relevant legislation. This will require a data processing agreement or similar with the recipient of the data. This procedure is in accordance

For peer review only - http://bmjopen.bmj.com/site/about/guidelines.xhtml

with the General Data Protection Regulation and relates to the high sensitivity of the data and the potential harm to the individual should the data be publicly known.

For peer review only

For peer review only - http://bmjopen.bmj.com/site/about/guidelines.xhtml

References

1. Chase JAD, Lozano A, Hanlon A, Bowles KH. Identifying Factors Associated With Mobility Decline Among Hospitalized Older Adults. Clin Nurs Res 2018;27(1):81-104.

2. Loyd C, Markland AD, Zhang Y, Fowler M, Harper S, Wright NC, et al. Prevalence of Hospital-Associated Disability in Older Adults: A Meta-analysis. J Am Med Dir Ass 2020;21(4):455-+.

3. Boyd CM, Landefeld CS, Counsell SR, Palmer RM, Fortinsky RH, Kresevic D, et al. Recovery of Activities of Daily Living in Older Adults After Hospitalization for Acute Medical Illness. J Am Ger Soc 2008;56(12):2171-9.

4. Brown CJ, Redden DT, Flood KL, Allman RM. The Underrecognized Epidemic of Low Mobility During Hospitalization of Older Adults. J Am Ger Soc 2009;57(9):1660-5.

5. Tanner RE, Brunker LB, Agergaard J, Barrows KM, Briggs RA, Kwon OS, et al. Age-related differences in lean mass, protein synthesis and skeletal muscle markers of proteolysis after bed rest and exercise rehabilitation. J Physiol 2015;593(18):4259-73.

6. Coker RH, Hays NP, Williams RH, Wolfe RR, Evans WJ. Bed Rest Promotes Reductions in Walking Speed, Functional Parameters, and Aerobic Fitness in Older, Healthy Adults. J Gerontol A Biol Sci Med Sci 2015;70(1):91-6.

7. Manas A, del Pozo-Cruz B, Rodriguez-Gomez I, Losa-Reyna J, Rodriguez-Manas L, Garcia-Garcia FJ, et al. Can Physical Activity Offset the Detrimental Consequences of Sedentary Time on Frailty? A Moderation Analysis in 749 Older Adults Measured With Accelerometers. J Am Med Dir Ass 2019;20(5):634-+.

8. Trevisan C, Vetrano DL, Calvani R, Picca A, Welmer AK. Twelve-year sarcopenia trajectories in older adults: results from a population-based study. J Cachexia Sarcopenia Muscle 2022;13(1):254-263.

For peer review only - http://bmjopen.bmj.com/site/about/guidelines.xhtml

9. Valenzuela PL, Morales JS, Castillo-Garcia A, Mayordomo-Cava J, Garcia-Hermoso A, Izquierdo M, et al. Effects of exercise interventions on the functional status of acutely hospitalised older adults: A systematic review and meta-analysis. Ageing Res Rev 2020;61.

10. Martinez-Velilla N, Casas-Herrero A, Zambom-Ferraresi F, de Asteasu MLS, Lucia A, Galbete A, et al. Effect of Exercise Intervention on Functional Decline in Very Elderly Patients During Acute Hospitalization A Randomized Clinical Trial. JAMA Intern Med 2019;179(1):28-36.

11. Ortiz-Alonso J, Bustamante-Ara N, Valenzuela PL, Vidan-Astiz M, Rodriguez-Romo G, Mayordomo-Cava J, et al. Effect of a Simple Exercise Program on Hospitalization-Associated Disability in Older Patients: A Randomized Controlled Trial. J Am Med Dir Ass 2020;21(4):531-+.

12. Levack WMM, Engkasan JP, Heinemann AW, Negrini S. A Review of CONSORT Guidelines About Comparison Groups With a Focused Discussion on Implications for Rehabilitation Clinical Trials. Am J Phys Med Rehabil 2020;99(3):191-7.

13. Pavon JM, Sloane RJ, Pieper CF, Colon-Emeric CS, Cohen HJ, Gallagher D, et al. Accelerometer-Measured Hospital Physical Activity and Hospital-Acquired Disability in Older Adults. J Am Ger Soc 2020;68(2):261-5.

14. Guralnik JM, Simonsick EM, Ferrucci L, Glynn RJ, Berkman LF, Blazer DG, et al. A short physical performance battery assessing lower-extremity function - association with self-reported disability and prediction of mortality and nursing-home admission. J Gerontol 1994;49(2):M85-M94.

15. Pavasini R, Guralnik J, Brown JC, di Bari M, Cesari M, Landi F, et al. Short Physical Performance Battery and all-cause mortality: systematic review and meta-analysis. BMC Med 2016;14.

For peer review only - http://bmjopen.bmj.com/site/about/guidelines.xhtml

16. Volpato S, Cavalieri M, Sioulis F, Guerra G, Maraldi C, Zuliani G, et al. Predictive Value of the Short Physical Performance Battery Following Hospitalization in Older Patients. J Gerontol A Biol Sci Med Sci 2011;66(1):89-96.

17. vanBennekom CAM, Jelles F, Lankhorst GJ, Bouter LM. Responsiveness of the Rehabilitation Activities Profile and the Barthel Index. J Clin Epidemiol 1996;49(1):39-44.

18. Herdman M, Gudex C, Lloyd A, Janssen MF, Kind P, Parkin D, et al. Development and preliminary testing of the new five-level version of EQ-5D (EQ-5D-5L). Qual Life Res 2011;20(10):1727-36.

19. Cruz-Jentoft AJ, Bahat G, Bauer J, Boirie Y, Bruyere O, Cederholm T, et al. Sarcopenia: revised European consensus on definition and diagnosis. Age Ageing 2019;48(1):16-31.

20. Rockwood K, Song XW, MacKnight C, Bergman H, Hogan DB, McDowell I, et al. A global clinical measure of fitness and frailty in elderly people. Can Med Ass J 2005;173(5):489-95.

21. Kaiser MJ, Bauer JM, Ramsch C, Uter W, Guigoz Y, Cederholm T, et al. Validation of the Mini Nutritional Assessment short-form (MNAA (R)-SF): A practical tool for identification of nutritional status. J Nutr Health Aging 2009;13(9):782-8.

22. Perera S, Mody SH, Woodman RC, Studenski SA. Meaningful change and responsiveness in common physical performance measures in older adults. J Am Ger Soc 2006;54(5):743-9.

23. De MNA, Keating JL, Jeffs K. Exercise for acutely hospitalised older medical patients. Cochrane Database Syst Rev 2007(1).

24. European Union. OE. Health at a Glance: Europe 2016. OECD Publishinghing 2016.

For peer review only

25 Reimers M, Eriksdotter M, Seiger A, Fastbom J. Prescription Changes During Geriatric Care Episodes: A Trend Analysis Conducted in Sweden. Drugs Aging 2018;35(3):243-8.

For peer review only

For peer review only - http://bmjopen.bmj.com/site/about/guidelines.xhtml

For peer review only

Figure 1. Flowchart of the study participants.

For peer review only - http://bmjopen.bmj.com/site/about/guidelines.xhtml

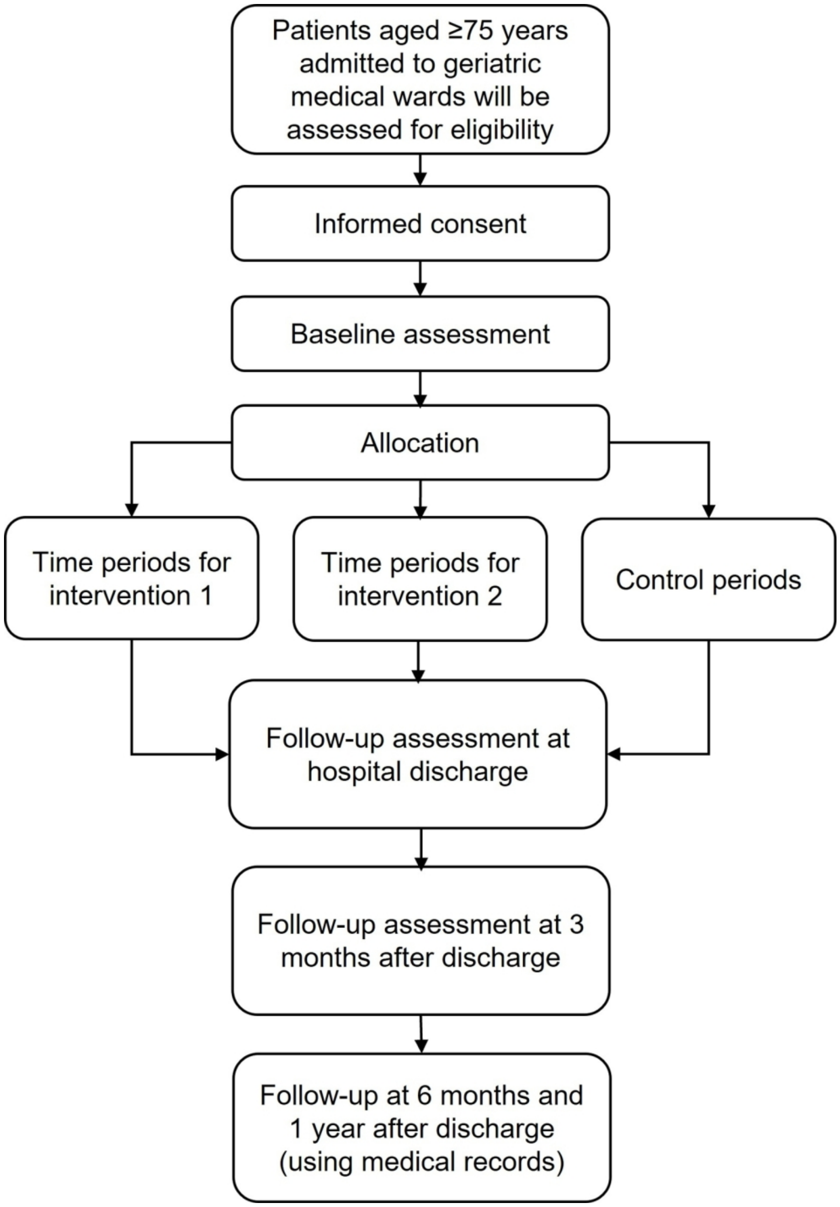

Figure 1. Flowchart of the study participants.

127x183mm (300 x 300 DPI)

STANDARD PROTOCOL ITEMS: RECOMMENDATIONS FOR INTERVENTIONAL TRIALS

SPIRIT 2013 Checklist: Recommended items to address in a clinical trial protocol and related documents*

Section/item	Item No	Description	Addressed on page number
**Administrative information**			
Title	1	Descriptive title identifying the study design, population, interventions, and, if applicable, trial acronym	Page 1, Title page
Trial registration	2a	Trial identifier and registry name. If not yet registered, name of intended registry	Page 3, Abstract
	2b	All items from the World Health Organization Trial Registration Data Set	
Protocol version	3	Date and version identifier	Page 10, Paragraph 3
Funding	4	Sources and types of financial, material, and other support	Page 14, Funding statement
Roles and responsibilities	5a	Names, affiliations, and roles of protocol contributors	Page 1, Title page and page 13, Authors' contributions
	5b	Name and contact information for the trial sponsor	Page 14, Funding statement
	5c	Role of study sponsor and funders, if any, in study design; collection, management, analysis, and interpretation of data; writing of the report; and the decision to submit the report for publication, including whether they will have ultimate authority over any of these activities	Page 14, Funding statement

For peer review only

	5d	Composition, roles, and responsibilities of the coordinating centre, steering committee, endpoint adjudication committee, data management team, and other individuals or groups overseeing the trial, if applicable (see Item 21a for data monitoring committee)	N/A
**Introduction**			
Background and rationale	6a	Description of research question and justification for undertaking the trial, including summary of relevant studies (published and unpublished) examining benefits and harms for each intervention	Page 5-6, Introduction
	6b	Explanation for choice of comparators	Page 5, Introduction, Paragraph 3
Objectives	7	Specific objectives or hypotheses	Page 6, Objectives
Trial design	8	Description of trial design including type of trial (eg, parallel group, crossover, factorial, single group), allocation ratio, and framework (eg, superiority, equivalence, noninferiority, exploratory)	Page 6, Trial design
**Methods: Participants, interventions, and outcomes**			
Study setting	9	Description of study settings (eg, community clinic, academic hospital) and list of countries where data will be collected. Reference to where list of study sites can be obtained	Page 6, Trial design
Eligibility criteria	10	Inclusion and exclusion criteria for participants. If applicable, eligibility criteria for study centres and individuals who will perform the interventions (eg, surgeons, psychotherapists)	Page 7, Inclusion and exclusion criteria, Page 7-8 Interventions
Interventions	11a	Interventions for each group with sufficient detail to allow replication, including how and when they will be administered	Page 7-8, Interventions, Page 19, Table 1

For peer review only - http://bmjopen.bmj.com/site/about/guidelines.xhtml

	11b	Criteria for discontinuing or modifying allocated interventions for a given trial participant (eg, drug dose change in response to harms, participant request, or improving/worsening disease)	N/A
	11c	Strategies to improve adherence to intervention protocols, and any procedures for monitoring adherence (eg, drug tablet return, laboratory tests)	Page 8, Paragraph 3
	11d	Relevant concomitant care and interventions that are permitted or prohibited during the trial	N/A
Outcomes	12	Primary, secondary, and other outcomes, including the specific measurement variable (eg, systolic blood pressure), analysis metric (eg, change from baseline, final value, time to event), method of aggregation (eg, median, proportion), and time point for each outcome. Explanation of the clinical relevance of chosen efficacy and harm outcomes is strongly recommended	Page 8-9, Data collection, Page 21, Table 2
Participant timeline	13	Time schedule of enrolment, interventions (including any run-ins and washouts), assessments, and visits for participants. A schematic diagram is highly recommended (see Figure)	Page 10, Trial status, Page 19, Figure 1
Sample size	14	Estimated number of participants needed to achieve study objectives and how it was determined, including clinical and statistical assumptions supporting any sample size calculations	Page 9, Data analysis
Recruitment	15	Strategies for achieving adequate participant enrolment to reach target sample size	Page 6, Trial design

Methods: Assignment of interventions (for controlled trials)

Allocation:

Sequence generation	16a	Method of generating the allocation sequence (eg, computer-generated random numbers), and list of any factors for stratification. To reduce predictability of a random sequence, details of any planned restriction (eg, blocking) should be provided in a separate document that is unavailable to those who enrol participants or assign interventions	Page 6, Trial design
Allocation concealment mechanism	16b	Mechanism of implementing the allocation sequence (eg, central telephone; sequentially numbered, opaque, sealed envelopes), describing any steps to conceal the sequence until interventions are assigned	Page 6, Trial design

Implementation	16c	Who will generate the allocation sequence, who will enrol participants, and who will assign participants to interventions	Page 6, Trial design
Blinding (masking)	17a	Who will be blinded after assignment to interventions (eg, trial participants, care providers, outcome assessors, data analysts), and how	Page 8, Data collection
	17b	If blinded, circumstances under which unblinding is permissible, and procedure for revealing a participant's allocated intervention during the trial	N/A

Methods: Data collection, management, and analysis

Data collection methods	18a	Plans for assessment and collection of outcome, baseline, and other trial data, including any related processes to promote data quality (eg, duplicate measurements, training of assessors) and a description of study instruments (eg, questionnaires, laboratory tests) along with their reliability and validity, if known. Reference to where data collection forms can be found, if not in the protocol	Page 9-10, Data collection
	18b	Plans to promote participant retention and complete follow-up, including list of any outcome data to be collected for participants who discontinue or deviate from intervention protocols	Page 9-10, Data collection, Secondary outcomes
Data management	19	Plans for data entry, coding, security, and storage, including any related processes to promote data quality (eg, double data entry; range checks for data values). Reference to where details of data management procedures can be found, if not in the protocol	Page 11, Ethics and dissemination
Statistical methods	20a	Statistical methods for analysing primary and secondary outcomes. Reference to where other details of the statistical analysis plan can be found, if not in the protocol	Page 9-10, Data analysis
	20b	Methods for any additional analyses (eg, subgroup and adjusted analyses)	Page 9-10, Data analysis
	20c	Definition of analysis population relating to protocol non-adherence (eg, as randomised analysis), and any statistical methods to handle missing data (eg, multiple imputation)	Page 9-10, Data analysis

Methods: Monitoring

Data monitoring	21a	Composition of data monitoring committee (DMC); summary of its role and reporting structure; statement of whether it is independent from the sponsor and competing interests; and reference to where further details about its charter can be found, if not in the protocol. Alternatively, an explanation of why a DMC is not needed	N/A
	21b	Description of any interim analyses and stopping guidelines, including who will have access to these interim results and make the final decision to terminate the trial	N/A
Harms	22	Plans for collecting, assessing, reporting, and managing solicited and spontaneously reported adverse events and other unintended effects of trial interventions or trial conduct	Page 8, Paragraph 3
Auditing	23	Frequency and procedures for auditing trial conduct, if any, and whether the process will be independent from investigators and the sponsor	Page 14, Funding statement
**Ethics and dissemination**			
Research ethics approval	24	Plans for seeking research ethics committee/institutional review board (REC/IRB) approval	Page 11, Ethics and dissemination
Protocol amendments	25	Plans for communicating important protocol modifications (eg, changes to eligibility criteria, outcomes, analyses) to relevant parties (eg, investigators, REC/IRBs, trial participants, trial registries, journals, regulators)	Page 10, Trial status
Consent or assent	26a	Who will obtain informed consent or assent from potential trial participants or authorised surrogates, and how (see Item 32)	Page 11, Ethics and dissemination
	26b	Additional consent provisions for collection and use of participant data and biological specimens in ancillary studies, if applicable	N/A
Confidentiality	27	How personal information about potential and enrolled participants will be collected, shared, and maintained in order to protect confidentiality before, during, and after the trial	Page 11, Ethics and dissemination

peer review only

For peer review only - http://bmjopen.bmj.com/site/about/guidelines.xhtml

Declaration of interests	28	Financial and other competing interests for principal investigators for the overall trial and each study site	Page 14, Competing interests
Access to data	29	Statement of who will have access to the final trial dataset, and disclosure of contractual agreements that limit such access for investigators	Page 11, Ethics and dissemination
Ancillary and post-trial care	30	Provisions, if any, for ancillary and post-trial care, and for compensation to those who suffer harm from trial participation	N/A
Dissemination policy	31a	Plans for investigators and sponsor to communicate trial results to participants, healthcare professionals, the public, and other relevant groups (eg, via publication, reporting in results databases, or other data sharing arrangements), including any publication restrictions	Page 11, Ethics and dissemination
	31b	Authorship eligibility guidelines and any intended use of professional writers	Page 11, Ethics and dissemination
	31c	Plans, if any, for granting public access to the full protocol, participant-level dataset, and statistical code	N/A
**Appendices**			
Informed consent materials	32	Model consent form and other related documentation given to participants and authorised surrogates	N/A
Biological specimens	33	Plans for collection, laboratory evaluation, and storage of biological specimens for genetic or molecular analysis in the current trial and for future use in ancillary studies, if applicable	N/A

*It is strongly recommended that this checklist be read in conjunction with the SPIRIT 2013 Explanation & Elaboration for important clarification on the items. Amendments to the protocol should be tracked and dated. The SPIRIT checklist is copyrighted by the SPIRIT Group under the Creative Commons "Attribution-NonCommercial-NoDerivs 3.0 Unported" license.

For peer review only - http://bmjopen.bmj.com/site/about/guidelines.xhtml

A study protocol for the "Preventing functional decline in acutely hospitalized older patients (PREV_FUNC)" study – Effects of two multicomponent exercise programs on physical function: a three-armed randomized controlled trial

Journal:	*BMJ Open*
Manuscript ID	bmjopen-2022-070885.R1
Article Type:	Protocol
Date Submitted by the Author:	19-May-2023
Complete List of Authors:	Welmer, Anna-Karin; Karolinska Institutet, Department of Neurobiology, Care Sciences and Society<br>Sandberg, Linda; Karolinska Institutet, Department of Neurobiology, Care Sciences and Society<br>Sandlund, Christina; Karolinska Institutet, Department of Neurobiology, Care Sciences and Society<br>Björck, Caroline; Uppsala University, Department of Women´s and Children´s Health<br>Hagströmer, Maria; Karolinska Institutet, Department of Neurobiology Care Sciences and<br>Hamilton, Julia; Sabbatsberg Hospital, Department of Geriatric Medicine<br>Helgstrand, Gunilla; Sabbatsberg Hospital, Department of Geriatric Medicine; Karolinska Institutet, Department of Neurobiology, Care Sciences and Society<br>Lindgren , Charlotte; Dalengeriatriken Aleris Närsjukvård AB, Department of Geriatric medicine<br>Nordstrand, Linda; Karolinska University Hospital; Karolinska Institutet, Department of Neurobiology, Care Sciences and Society<br>Sandstedt, Petter; Karolinska University Hospital<br>Kivipelto, Miia; Karolinska Institutet, Division of Geriatric Epidemiology<br>Boström, Anne-Marie; Karolinska Institutet, Department of Neurobiology, Care Sciences and Society; Karolinska University Hospital, Theme Inflammation and Aging
**Primary Subject Heading**:	Geriatric medicine
Secondary Subject Heading:	Rehabilitation medicine
Keywords:	GERIATRIC MEDICINE, PREVENTIVE MEDICINE, Clinical trials < THERAPEUTICS

SCHOLARONE™ Manuscripts

A study protocol for the "Preventing functional decline in acutely hospitalized older patients (PREV_FUNC)" study – Effects of two multicomponent exercise programs on physical function: a three-armed randomized controlled trial

Anna-Karin Welmer[1,2,3], Linda Sandberg[1,4], Christina Sandlund[1,5,6], Caroline Björck[7,8,9], Maria Hagströmer[1,6], Julia Hamilton[10], Gunilla Helgstrand[10,11], Charlotte Lindgren[4], Linda Nordstrand[3,11], Petter Sandstedt[3], Miia Kivipelto[12,13,14], Anne-Marie Boström[13,14,15]

[1] Division of Physiotherapy, Department of Neurobiology, Care Sciences and Society, Karolinska Institutet, Stockholm, Sweden

[2] Aging Research Center, Department of Neurobiology, Care Sciences and Society, Karolinska Institutet and Stockholm University, Stockholm, Sweden

[3] Women´s Health and Allied Health Professionals Theme, Medical Unit Medical Psychology, Karolinska University Hospital, Stockholm, Sweden

[4] Department of Geriatric Medicine, Dalengeriatriken Aleris Närsjukvård AB, Stockholm, Sweden

[5] Division of Family Medicine and Primary Care, Department of Neurobiology, Care Sciences and Society, Karolinska Institutet, Stockholm, Sweden

[6] Academic Primary Health Care Centre, Region Stockholm, Stockholm, Sweden

[7] Department of Women´s and Children´s Health, Uppsala University, Uppsala, Sweden

[8] Centre for Research and Development, County Council of Gävleborg, Gävle, Sweden

[9] Department of Caring Sciences, University of Gävle, Gävle, Sweden

[10] Department of Geriatric Medicine, Sabbatsberg Hospital, Region Stockholm, Stockholm, Sweden

For peer review only

For peer review only - http://bmjopen.bmj.com/site/about/guidelines.xhtml

[11] Division of Occupational Therapy, Department of Neurobiology, Care Sciences and Society, Karolinska Institutet, Stockholm, Sweden

[12] Division of Clinical Geriatrics, Department of Neurobiology, Care Sciences and Society, Karolinska Institutet, Stockholm, Sweden

[13] Theme Inflammation and Aging, Medical Unit Aging, Karolinska University Hospital, Huddinge, Sweden

[14] Research and Development Unit, Stockholm´s Sjukhem, Stockholm, Sweden

[15] Division of Nursing, Department of Neurobiology, Care Science and Society, Karolinska Institutet, Huddinge, Sweden

Correspondence to:

Anna-Karin Welmer, PhD, Associate Professor

Karolinska Institutet

Department of Neurobiology, Care Sciences and Society

Division of Physiotherapy 23100

SE-141 83 Huddinge, Sweden

Email: anna-karin.welmer@ki.se

Phone: +46 8 524 888 04

For peer review only

Abstract

Introduction: Acutely hospitalized older patients often live with frailty and have an increased risk of impaired physical function. Previous studies suggest that exercise might mitigate the risk of physical impairment; however, further research is needed to compare the effect of different types of exercise interventions. In this paper we report a protocol for a trial that aims to examine 1) if multicomponent exercise interventions (interventions that include both mobility and strengthening exercises) have effects on physical function compared to usual care in older adults, and 2) if a comprehensive multicomponent exercise program is more effective than a simple multicomponent exercise program that only include walking and sit-to-stand exercises.

Methods and analysis: This is a three-armed randomized controlled trial, with two intervention groups (comprehensive and simple exercise program) and a control group receiving usual care. We will include 320 participants aged ≥75 years from geriatric medical departments of four hospitals in Stockholm, Sweden. Assessments will be conducted at hospital admission, discharge and three months thereafter concerning physical function (primary outcome), activities of daily living, health-related quality of life, sarcopenia, and falls. The number of re-admissions will be registered up to 1 year after discharge. Data will be analyzed with linear mixed effects models, according to the intention-to-treat approach.

Ethics and dissemination: Ethical approval for this trial has been granted by the Swedish Ethical Review Authority (approval number 2022-03032-01). Data collection will consider the information requirement, the requirement of consent, confidentiality obligations, and the utilization requirement. Trial findings will be disseminated through multiple channels, including scientific publications and conferences, and workshops with healthcare professionals and the public.

Trial registration: NCT05366075, ClinicalTrials.gov, 4 May 2022, https://clinicaltrials.gov/

For peer review only - http://bmjopen.bmj.com/site/about/guidelines.xhtml

Strengths and limitations of this trial

• In order for the sample to be representative of older hospitalized patients, the participants will be consecutively recruited at several hospitals in Stockholm

• Both exercise interventions use none or low technology exercise equipment that is already available in most hospital settings or can be purchased at a low price

• The block design may result in selection bias in the trial; however, one advantage is that participants from both groups at the same department are avoided

Keywords: Exercise**,** Hospitalization, Older Adults, Physical Functional Performance, Randomized Controlled Trial

Word count: 3875 words

For peer review only

For peer review only - http://bmjopen.bmj.com/site/about/guidelines.xhtml

Introduction

Hospitalization due to acute medical illness is associated with several negative health consequences in older adults, even if the illness that caused the admission is successfully treated. Approximately, half of adults aged 65 years and older show a decline in physical function following acute hospitalization (1). Many patients do not regain pre-hospital function, lose independence, and have increased risk of reduced well-being, readmissions, and death after discharge (2, 3).

Acutely hospitalized older patients often live with frailty and several concurrent diseases (3). Furthermore, acutely hospitalized older people spend most of their hospital stay in bed, even if they are able to walk independently (4). Such periods of physical inactivity, albeit short, may exacerbate the age-related loss of muscle mass, strength, and physical function (5, 6). It has been suggested that relatively small amounts of exercise might mitigate these deficits (7, 8), emphasizing the importance of identifying exercise interventions that are easy to use for older people during hospital stay.

A recent Cochrane review concluded that there is uncertainty regarding the effect of exercise interventions on functional outcomes for older medical inpatients during acute hospitalization (9). Thus, it has been suggested that future research on this topic should focus on consistent reporting of participant characteristics, including baseline level of functional ability, as well as exercise dose and adherence to provide an insight into the reasons for the observed inconsistencies in findings (9).

In a randomized controlled trial (RCT), Martinez-Velilla et al. demonstrated that a comprehensive exercise program, that consisted of several supervised exercises including progressive resistance, balance, and walking exercise, provided significant health benefits over usual care in acutely hospitalized patients aged 75 years and older (10). Also, in another RCT, Ortiz-Alonso et al. showed that a relatively simple multicomponent intervention solely

For peer review only - http://bmjopen.bmj.com/site/about/guidelines.xhtml

consisting of walking and rising from a chair decreased the risk of dependence in activities of daily living (ADL) in acutely hospitalized patients older than 75 years (11). It is not known whether a simple exercise program, such as the one by Ortiz-Alonso et al. could yield similar health benefits as a more comprehensive intervention, such as the one by Martinez-Velilla et al. Considering the time constraints in acute care settings, a simple exercise program may be more feasible if proven effective.

The main aims of the "Preventing functional decline in acutely hospitalized older patients (PREV_FUNC)" study are to 1) evaluate the effect of multicomponent exercise interventions (interventions that include both mobility and strengthening exercises) on physical function (primary outcome), ADL, health-related quality of life (HRQL), sarcopenia, falls, readmissions, and mortality in comparison with usual care in acutely hospitalized people aged 75 years and older, and 2) if a comprehensive multicomponent exercise program that include several exercises is more effective than a simple multicomponent exercise program that only include walking and sit-to-stand exercises. An initial pilot of the PREV_FUNC study suggested that the intervention was feasible, safe, and acceptable to the participants (unpublished data). In this paper we present a study protocol for the PREV_FUNC study.

Methods and analysis

Trial design

The PREV_FUNC study will be conducted in accordance with the Consolidated Standards of Reporting Trials (12). It is a three-armed single blind block randomized controlled multi-center trial. Participants will be included consecutively from geriatric medical departments at four hospitals in Stockholm, Sweden. A flowchart of the participants is shown in Figure 1. The patients will be included in the control group or one of the intervention groups in a time-dependent manner: participants will be recruited to each group during blocks of two to three

For peer review only - http://bmjopen.bmj.com/site/about/guidelines.xhtml

weeks, that will be repeated until the end of the trial. The order of the groups will be randomized for each trial site (randomization will be done by the responsible researchers). The block design has been chosen to avoid co-presence of participants from intervention and control groups at the same department; that is, to prevent the exercises in the intervention groups from spreading to usual treatment and risk contaminating the control group (11, 12). Eligible subjects will be invited to participate in the trial within 36 hours of admission and the baseline examination will be conducted directly after the inclusion. The trial has been registered at clinicaltrials.gov, NCT05366075. This protocol is reported in accordance with the Standard Protocol Items: Recommendations for Interventional Trials (13).

Inclusion and exclusion criteria

Inclusion criteria for participation are age 75 years and older, ability to stand up from a sitting position independently or with minimal personal help, and ability to communicate and collaborate with the research staff. People who cannot follow instructions will be excluded. Patients who by the responsible physician are assessed as not eligible to participate due to terminal illness or any major medical condition that contraindicates exercise will also be excluded. We will also exclude patients who live in nursing homes, or those who were previously included in the trial.

Interventions

The interventions will start the same day as the baseline examination or the day after and will be performed on all weekdays until discharge. *Intervention 1* is adapted from the exercise program by Ortiz-Alonso et al. (11), and *Intervention 2* is adapted from the exercise program by Martinez-Velilla et al. (14). The interventions will be offered in addition to usual care.

Intervention 1 (simple exercise program) (11). This intervention will include up to four sessions per day (total duration 20-30 minutes/day). It consists of sit-to stand exercises and walking along the corridor of the ward (for those who can walk). The exercises will mainly be

For peer review only - http://bmjopen.bmj.com/site/about/guidelines.xhtml

led by physiotherapists, and, when suitable, *Intervention 1* will be performed in connection with regular routines in the usual care (Table 1). At discharge, the participants will be encouraged to continue with the exercise program at home, three days per week during six weeks. In addition to oral instructions, they will receive written instructions with pictures on how to perform the exercise.

Intervention 2 (comprehensive exercise program) (14). This intervention consists of two daily sessions (morning and afternoon) with a duration of 20 minutes each. The morning session includes individualized supervised progressive resistance, balance, and walking exercise, tailored to each participant's capacity. The resistance exercise includes using weight cuffs, which mainly involves lower-extremity muscles. Balance and gait exercises include different exercises such as line walking, stepping practice, and walking with small obstacles. The evening session consists of functional exercises using light loads, such as knee extension and flexion, hand exercise with a ball, and daily walking exercise (for those who can walk). The exercises will be led by physiotherapists (Table 1).

Control group. The control group will receive usual care. Usual care is based on teamwork, which includes physiotherapy. The physiotherapy focuses on mobility assessments, and the patients may receive a limited amount of functional training when needed. However, we expect that the participants often have a limited amount of physical activity during the hospital stay (15).

Daily logbooks will be used during the hospital stay to register the number of attended exercise sessions for the intervention groups, as well as treatment safety for all groups (by registering adverse events during the hospital stay, e.g., falls). Furthermore, exercise diaries will be used during six weeks to register the self-training conducted at home in *Intervention 1*.

For peer review only - http://bmjopen.bmj.com/site/about/guidelines.xhtml

Table 1. Overview of the interventions.

Intervention	Timing/frequency and duration of exercises	Description	Dose
**Intervention 1**<br>(Simple exercise program)	Up to 4 times/day<br>(Total duration 20-30 minutes/day)	• **Sit-to-stand exercises.** Rising from a chair and sitting down again repeatedly, using armrest and assistance if needed<br>• **Walking.** Walking along the corridor, using walking aid and assistance if needed	1-3 sets of up to 10 repetitions, depending on the participant's ability<br>3-10 min per session, with resting periods if needed
**Intervention 2**<br>(Comprehensive exercise program)	Before lunch<br>(Duration 20 minutes)	*Strengthening exercises*<br>• **Sit-to-stand exercises.** Rising from a chair and sitting down again repeatedly, using armrest and assistance if needed<br>• **Knee extensions.** Knee extensions in a sitting position, with a weight cuff on the ancle. Repeat with both legs<br>• **Standing push-ups.** Bending and stretching arms leaning on a wall	1-3 sets of up to 10 repetitions, depending on the participant's ability
		Balance exercises<br>• **Semi-tandem stance.** One foot placed in front of the other foot, with the big toe of the back foot in the groove of the front foot. Repeat on the other side<br>• **Line walking.** Walking in a straight line, placing one foot in front of the other, and vice versa<br>• **Walking with small obstacles**. Walking around small obstacles and/or stepping over small obstacles<br>• **Stepping practice**. Stepping up and down on a step board or staircase<br>• **Weight transfer.** Standing up, transferring the weight from one leg to the other<br>• **Proprioceptive exercises**. Walking on an unstable surface	Dose adapted to the participant's ability and time frame
	Afternoon	• **Knee extensions.** Knee extensions in a sitting position. Repeat with both legs<br>• **Knee flexion.** Standing knee flexions, holding on to a chair. Repeat with both legs	1-3 sets of up to 10 repetitions for, depending on the participant's ability

For peer review only - http://bmjopen.bmj.com/site/about/guidelines.xhtml

	(Duration 20 minutes)	• **Hip abduction.** Standing hip abduction with the participant standing, holding on to a chair. Repeat with both legs<br>• **Hand exercise** with handgrip ball<br>• **Walking.** Walking along the corridor, using walking aid and assistance if needed	

For peer review only

For peer review only - http://bmjopen.bmj.com/site/about/guidelines.xhtml

Data collection

Physical function, ADL, HRQL, sarcopenia and falls will be assessed at baseline (hospital admission), discharge, and three months after discharge. Research staff blinded to group allocation will conduct the tests and administer the questionnaires at the hospital (for the baseline and discharge evaluations) and at the home of the participants (for the three-month evaluation). The six-month and the one-year evaluation will be conducted using patient records (for hospital readmissions and mortality) (Table 2).

The primary outcome will be lower extremity physical function, assessed according to the Short Physical Performance Battery (SPPB) (16). The SPPB has three components: standing balance, walking speed, and the chair stand test for lower extremity strength. It has been validated in older adults living with frailty, is sensitive to change, and predicts a wide range of clinical outcomes (17, 18).

Secondary outcomes will be ADL (according to the Barthel Index) (19), HRQL (according to the EuroQol–5 Dimension) (20), presence of sarcopenia (defined by handgrip strength, calf circumference, and arm circumference), according to the European Working Group's revised criteria on Sarcopenia in Older People (21), and number of falls during follow-up (according to the logbooks at the hospital, and self-report at the three-month follow-up). Data on vital status, and number of readmissions up to one year after discharge will be collected using patient records.

Baseline data will also be collected from patient records including demographic data (age, sex, and cohabitation status), reason for hospital admission, comorbidities, frailty status, and nutritional risk. Disease diagnoses (reason for hospital admission and comorbidities) will be coded based on the International Classification of Diseases Tenth Revision (ICD-10), and further operationalized using a previously described methodology (22). Frailty status will be defined according to the Clinical Frailty Scale (23), and nutritional risk will be defined

For peer review only - http://bmjopen.bmj.com/site/about/guidelines.xhtml

according to the Mini Nutritional Assessment-Short Form (24). Type and amount of nutritional supplementation consumed during the hospitalization (if applicable) will be recorded in a logbook. Falls in the previous year will be assessed according to self-report at baseline. Length of hospital stay, and discharge destination will be registered at discharge.

For peer review only

For peer review only - http://bmjopen.bmj.com/site/about/guidelines.xhtml

Table 2. Time of assessment of the different variables in the trial.

	Time point				
**Assessment**	Baseline (hospital admission)	After exercise/control period (discharge)	3 months after discharge	6 months after discharge	1 year after discharge
**Primary outcome**					
Physical function	X	X	X		
**Secondary outcomes**					
Activities of daily living	X	X	X		
Health-related quality of life	X	X	X		
Sarcopenia	X	X	X		
Falls after inclusion		X	X		
Hospital readmissions			X	X	X
Mortality			X	X	X
**Additional data**					
Frailty status	X				
Nutritional risk	X				
Nutritional supplementation	Continuously collected during the hospitalization				
Falls in the previous year	X				
Demographic data	X				
Reason for hospital admission	X				
Comorbidities	X				
Length of hospital stay		X			
Discharge destination		X			

For peer review only - http://bmjopen.bmj.com/site/about/guidelines.xhtml

Data analysis

Power of the trial. We estimate that 264 participants should be included in the main RCT to enable detection of a clinically meaningful difference of one point in the main outcome (SPPB) at three months after discharge (25), when power is 0.80 and alpha is 0.05 (three group comparison). To take into account approximately 20 percent deaths or dropouts during three months, 320 participants should be included in the trial.

We will use the intention-to-treat approach. Between-group comparisons of change in the continuous variables from baseline to follow-up will be conducted using linear mixed effects models, with adjustments for baseline scores. To account for deaths and loss to follow-up, we will model the group comparisons through joint models, which will be performed by simultaneously estimating both a linear mixed model and a Cox's proportional hazard regression model. Group differences in the number of readmissions will be examined using ordinal logistic regression models. Binary logistic regression models will be employed to examine between group differences in binary outcomes, and Cox's proportional hazard regression model will be used for mortality data. Multiplicative interactions between sex and group allocation in relation to the outcomes will also be examined. Statistical analyses will be performed with Stata and R.

Patient and public involvement

The trial is conducted in dialog between researchers, clinicians, and health care managers of different disciplines. Although the interventions will mainly be led by physiotherapists, an intervention at a geriatric department requires a dialog with several professions to meet the needs of the older adults, who often have several concurrent health problems. Thus, the trial has an interdisciplinary and practice-oriented focus, which will facilitate future implementation if the interventions are proven to be effective. Furthermore, qualitative interviews will be conducted among the participants in the two intervention groups, to explore

For peer review only

For peer review only - http://bmjopen.bmj.com/site/about/guidelines.xhtml

their experiences of the interventions. The interviews will be conducted at the hospital during the hospital stay, or at the participant's home after discharge.

Trial status

This is version 1 of the study protocol. Recruitment for the trial is ongoing and is planned between September 2022 and December 2023. Any deviations to this protocol will be updated on clinicaltrials.gov, and the changes will be reported when disseminating the results.

Ethics and dissemination

Ethical approval for this trial has been granted by the Swedish Ethical Review Authority (approval number 2022-03032-01). Data collection will consider the information requirement, the requirement of consent, confidentiality obligations, and the utilization requirement. The participants will receive verbal and written information, and informed written consent will be collected by research staff (supplementary file). All data collected is pseudonymized and stored in paper and digital format in accordance with the General Data Protection Regulation. Only the responsible researchers will have full access to the data. Trial findings will be disseminated through multiple channels, including scientific publications and conferences, and workshops with healthcare professionals and the public. We will follow the Vancouver recommendations for authorship.

Discussion

The goal of this trial is to provide knowledge on how to prevent physical impairment in acutely hospitalized patients aged 75 years and older. Although several studies have shown beneficial effects of community-based exercise programs in older adults, less attention have been given to exercise programs during hospitalization for acute medical illness (26). To date,

For peer review only - http://bmjopen.bmj.com/site/about/guidelines.xhtml
For peer review only

there is no consensus concerning the most effective exercise intervention to prevent functional decline in acutely hospitalized older adults (14).

Older adults consume a large proportion of health care resources (27). Current guidelines for care of various diseases are mainly based on research performed on younger people without severe comorbidity and cannot always be generalized to older patients. This trial may provide a novel strategy to maintain or improve physical function in this group of older adults living with frailty. If the trial will show positive effects, we plan to further study its implementation and analyze its cost-effectiveness in subsequent studies. If our results are positive, it might lead to a change in clinical practice where multicomponent exercises are incorporated in routine care. Our outcome, physical function, is an important component of healthy aging and a strong predictor of future health status and mortality following hospitalization in older adults (17, 18).

Our interventions are based on two previous studies conducted among Spanish hospitalized older adults (10, 11). The results from these studies indicate that multicomponent exercises are safe and effective in counteracting functional limitations in older hospitalized people. Research is however needed on the generalizability of these approaches to other settings. Furthermore, it is not known whether these approaches are equally efficient in counteracting functional limitations in older hospitalized people. In the present trial, we have modified the intervention by Martinez-Velilla et al. (10), by using weight cuffs instead of resistance exercise machines. This adaption was done to enhance future implementation in clinical settings. The exercise interventions examined in this trial use either no exercise equipment or low technology exercise equipment that is already available in most hospital settings or can be purchased at a low cost. Furthermore, we modified the intervention by Ortiz-Alonso et al. by increasing the total duration of the exercise from 20 minutes per day to 20-30 minutes per day (11). This was done to make the two interventions more comparable in terms of daily exercise

For peer review only - http://bmjopen.bmj.com/site/about/guidelines.xhtml

time during the hospital stay. Finally, the interventions will only be performed on weekdays to fit with staff resources during weekends.

We expect that this trial will help identify the exercise program that is most effective and therefore may be most suitable for future implementation. The two exercise programs have different possible strengths and weaknesses. For instance, it may be hypothesized that the comprehensive exercise program may have a better effect on physical function at hospital discharge since it is more individualized and intensive than the simple exercise program. However, a disadvantage is that it is less suitable for self-training than the simple exercise program, due to its complexity and the possible risk entailed by doing unsupervised balance training. Thus, it may be hypothesized that the simple exercise program, which is more suitable for self-training, can have an equal or better effect on physical function than the comprehensive exercise program three months after discharge since the participants can continue with the program at home. Furthermore, in future studies we plan to conduct post-hoc analyses to identify associations between baseline factors, e.g., frailty level, and the effect of the intervention.

This trial has some limitations. First, instead of a classic randomization we have chosen a block design, which may introduce selection bias in the trial. However, an advantage of the block design is that it avoids co-presence of participants from both groups at the same department; and thus, prevents that the exercises in the intervention groups will spread to ordinary treatment and risk to contaminate the control group. If the exercises in the intervention groups would spread to the control group and produce unexpected clinical benefits, it could create a bias by disguising a possible genuine benefit of the interventions and reduce the effect size to a value that is not clinically significant. Therefore, it has been emphasized that researchers in clinical trials on rehabilitation topics should make sure that control groups do not include any of the active elements of the intervention such as physical

For peer review only - http://bmjopen.bmj.com/site/about/guidelines.xhtml

For peer review only

exercise (12). Second, like usual care in the studies by Martinez-Velilla et al. and Ortiz-Alonso et al. (10, 11), usual care at geriatric departments in Sweden include physiotherapy, and the patients may receive a limited amount of functional training when needed. This may introduce a bias in the trial since participants in the control group may also receive some physical exercise. However, a hospital stay is often accompanied by low levels of physical activity, which has been suggested to play a major role in causing the negative health consequences associated with hospitalization (4, 15). Third, the trial will include participants who are able to stand up from a sitting position, and to communicate and collaborate with the research team, which could limit the generalizability to those with relatively higher levels of functioning. However, the trial participants will be consecutively recruited at several hospitals, so that the sample will be representative of older hospitalized patients.

A possible challenge in this trial may be the gradual change towards shortened length of hospital stay for geriatric inpatients in Sweden (28). A further shortened average length of hospital stay may limit the possibilities to show significant effects of the interventions during the hospital stay. Thus, in *Intervention 1*, the participants will be encouraged to continue with the exercises at home. In future studies, we plan to explore prerequisites for a transitional care intervention, in which physical exercise is performed during acute hospitalization and as self-training at home with support from primary care. This three-armed intervention is a first step towards finding the best strategies to maintain or improve physical function among acutely hospitalized older adults.

In summary, the PREV_FUNC trial has the potential to provide new knowledge about how to maintain and improve physical function in acutely hospitalized older patients. The number of hospital admissions is likely to increase as the population ages. Identifying the most effective interventions to reduce health consequences of acute hospitalization in older adults is thus highly relevant from clinical and public health perspectives.

For peer review only - http://bmjopen.bmj.com/site/about/guidelines.xhtml

Author contributions

AKW, AMB and LS designed the protocol and methods of the trial. AKW, LS, CS, CB, MH, JH, GH, CL, LN, PS, MK and AMB contributed with the writing of the manuscript. All authors read and approved the final manuscript.

Funding statement

The PREV_FUNC trial is supported by grants provided by the Swedish Research Council for Health, Working Life and Welfare, Grant Number 2021-01788; Region Stockholm (ALF project), Grant Number FoUI-960460, Center for Innovative Medicine (CIMED), Region Stockholm, Grant Number FoUI-961330; and Konung Gustaf V:s och Drottning Victorias Frimurarestiftelse (PI AKW). In addition, MK was supported by Stiftelsen Stockholms Sjukhem, CIMED and the Swedish Research Council, Grant Number 2017-0610. The funders have no role in design of the trial, collection, management, analysis, interpretation of data, writing manuscripts, or in the decision to submit for publication.

Competing interests

The authors declare that they have no competing interests.

Data statement

Data sharing is not applicable to this article as no datasets were generated or analyzed during the current trial. The data that will be collected will be available upon request; requests for access to the data can be put to the Research Data Office (rdo@ki.se) at Karolinska Institutet and will be handled according to the relevant legislation. This will require a data processing agreement or similar with the recipient of the data. This procedure is in accordance with the

For peer review only - http://bmjopen.bmj.com/site/about/guidelines.xhtml

General Data Protection Regulation and relates to the high sensitivity of the data and the potential harm to the individual if the data would be publicly known.

For peer review only

For peer review only - http://bmjopen.bmj.com/site/about/guidelines.xhtml

References

1. Chase JAD, Lozano A, Hanlon A, Bowles KH. Identifying Factors Associated With Mobility Decline Among Hospitalized Older Adults. Clin Nurs Res 2018;27(1):81-104.

2. Loyd C, Markland AD, Zhang Y, Fowler M, Harper S, Wright NC, et al. Prevalence of Hospital-Associated Disability in Older Adults: A Meta-analysis. J Am Med Dir Ass 2020;21(4):455-+.

3. Boyd CM, Landefeld CS, Counsell SR, Palmer RM, Fortinsky RH, Kresevic D, et al. Recovery of Activities of Daily Living in Older Adults After Hospitalization for Acute Medical Illness. J Am Ger Soc 2008;56(12):2171-9.

4. Brown CJ, Redden DT, Flood KL, Allman RM. The Underrecognized Epidemic of Low Mobility During Hospitalization of Older Adults. J Am Ger Soc 2009;57(9):1660-5.

5. Tanner RE, Brunker LB, Agergaard J, Barrows KM, Briggs RA, Kwon OS, et al. Age-related differences in lean mass, protein synthesis and skeletal muscle markers of proteolysis after bed rest and exercise rehabilitation. J Physiol 2015;593(18):4259-73.

6. Coker RH, Hays NP, Williams RH, Wolfe RR, Evans WJ. Bed Rest Promotes Reductions in Walking Speed, Functional Parameters, and Aerobic Fitness in Older, Healthy Adults. J Gerontol A Biol Sci Med Sci 2015;70(1):91-6.

7. Manas A, del Pozo-Cruz B, Rodriguez-Gomez I, Losa-Reyna J, Rodriguez-Manas L, Garcia-Garcia FJ, et al. Can Physical Activity Offset the Detrimental Consequences of Sedentary Time on Frailty? A Moderation Analysis in 749 Older Adults Measured With Accelerometers. J Am Med Dir Ass 2019;20(5):634-+.

8. Trevisan C, Vetrano DL, Calvani R, Picca A, Welmer AK. Twelve-year sarcopenia trajectories in older adults: results from a population-based study. J Cachexia Sarcopenia Muscle 2022;13(1):254-263.

For peer review only

9. Hartley P, Keating JL, Jeffs KJ, Raymond MJM, Smith TO. Exercise for acutely hospitalised older medical patients. Cochrane Database Syst Rev 2022(11).

10. Martinez-Velilla N, Casas-Herrero A, Zambom-Ferraresi F, de Asteasu MLS, Lucia A, Galbete A, et al. Effect of Exercise Intervention on Functional Decline in Very Elderly Patients During Acute Hospitalization A Randomized Clinical Trial. JAMA Intern Med 2019;179(1):28-36.

11. Ortiz-Alonso J, Bustamante-Ara N, Valenzuela PL, Vidan-Astiz M, Rodriguez-Romo G, Mayordomo-Cava J, et al. Effect of a Simple Exercise Program on Hospitalization-Associated Disability in Older Patients: A Randomized Controlled Trial. J Am Med Dir Ass 2020;21(4):531-+.

12. Levack WMM, Engkasan JP, Heinemann AW, Negrini S. A Review of CONSORT Guidelines About Comparison Groups With a Focused Discussion on Implications for Rehabilitation Clinical Trials. Am J Phys Med Rehabil 2020;99(3):191-7.

13. Chan AW, Tetzlaff JM, Altman DG, Dickersin K, Moher D. SPIRIT 2013: new guidance for content of clinical trial protocols. Lancet 2013;381(9861):91-2.

14. Valenzuela PL, Morales JS, Castillo-Garcia A, Mayordomo-Cava J, Garcia-Hermoso A, Izquierdo M, et al. Effects of exercise interventions on the functional status of acutely hospitalised older adults: A systematic review and meta-analysis. Ageing Res Rev 2020;61.

15. Pavon JM, Sloane RJ, Pieper CF, Colon-Emeric CS, Cohen HJ, Gallagher D, et al. Accelerometer-Measured Hospital Physical Activity and Hospital-Acquired Disability in Older Adults. J Am Ger Soc 2020;68(2):261-5.

16. Guralnik JM, Simonsick EM, Ferrucci L, Glynn RJ, Berkman LF, Blazer DG, et al. A short physical performance battery assessing lower-extremity function - association with

For peer review only - http://bmjopen.bmj.com/site/about/guidelines.xhtml

self-reported disability and prediction of mortality and nursing-home admission. J Gerontol 1994;49(2):M85-M94.

17. Pavasini R, Guralnik J, Brown JC, di Bari M, Cesari M, Landi F, et al. Short Physical Performance Battery and all-cause mortality: systematic review and meta-analysis. BMC Med 2016;14.

18. Volpato S, Cavalieri M, Sioulis F, Guerra G, Maraldi C, Zuliani G, et al. Predictive Value of the Short Physical Performance Battery Following Hospitalization in Older Patients. J Gerontol A Biol Sci Med Sci 2011;66(1):89-96.

19. vanBennekom CAM, Jelles F, Lankhorst GJ, Bouter LM. Responsiveness of the Rehabilitation Activities Profile and the Barthel Index. J Clin Epidemiol 1996;49(1):39-44.

20. Herdman M, Gudex C, Lloyd A, Janssen MF, Kind P, Parkin D, et al. Development and preliminary testing of the new five-level version of EQ-5D (EQ-5D-5L). Qual Life Res 2011;20(10):1727-36.

21. Cruz-Jentoft AJ, Bahat G, Bauer J, Boirie Y, Bruyere O, Cederholm T, et al. Sarcopenia: revised European consensus on definition and diagnosis. Age Ageing 2019;48(1):16-31.

22. Calderon-Larranaga A, Vetrano DL, Onder G, Gimeno-Feliu LA, Coscollar-Santaliestra C, Carfi A, et al. Assessing and Measuring Chronic Multimorbidity in the Older Population: A Proposal for Its Operationalization. J Gerontol A Biol Sci Med Sci 2016;72(10):1417–23.

23. Rockwood K, Song XW, MacKnight C, Bergman H, Hogan DB, McDowell I, et al. A global clinical measure of fitness and frailty in elderly people. Can Med Ass J 2005;173(5):489-95.

For peer review only - http://bmjopen.bmj.com/site/about/guidelines.xhtml

24. Kaiser MJ, Bauer JM, Ramsch C, Uter W, Guigoz Y, Cederholm T, et al. Validation of the Mini Nutritional Assessment short-form (MNAA (R)-SF): A practical tool for identification of nutritional status. J Nutr Health Aging 2009;13(9):782-8.

25. Perera S, Mody SH, Woodman RC, Studenski SA. Meaningful change and responsiveness in common physical performance measures in older adults. J Am Ger Soc 2006;54(5):743-9.

26. De MNA, Keating JL, Jeffs K. Exercise for acutely hospitalised older medical patients. Cochrane Database Syst Rev 2007(1).

27. European Union. OE. Health at a Glance: Europe 2016. OECD Publishinghing 2016.

28. Reimers M, Eriksdotter M, Seiger A, Fastbom J. Prescription Changes During Geriatric Care Episodes: A Trend Analysis Conducted in Sweden. Drugs Aging 2018;35(3):243-8.

For peer review only

For peer review only - http://bmjopen.bmj.com/site/about/guidelines.xhtml

For peer review only

Figure 1. Flowchart of the participants.

For peer review only - http://bmjopen.bmj.com/site/about/guidelines.xhtml

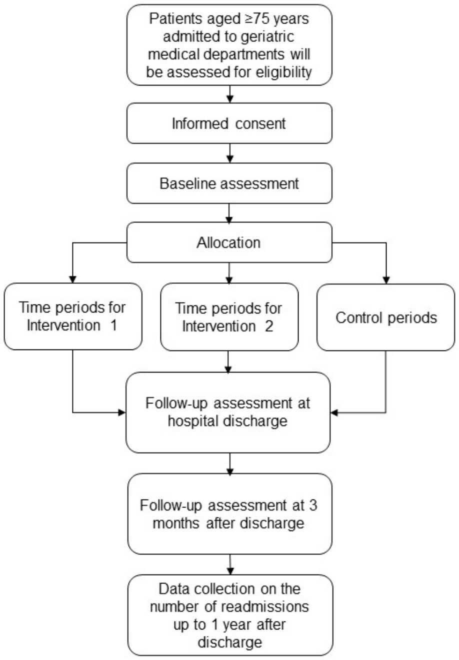

Figure 1. Flowchart of the participants.

190x338mm (96 x 96 DPI)

The “Preventing functional decline in acutely hospitalized older patients (PREV_FUNC)” study

Information about participation in research project

We would like to ask you if you would like to participate in a research project. In this document you will receive information about the project and what it means to participate.

What kind of project is it and why do you want me to participate?

Physical activity is important for good health. According to research, physical exercise during hospitalization can be effective in improving independence and function. The aim is therefore to explore the effect on physical function of two different exercise interventions during and after hospital stay compared to usual care. To participate, you must be 75 years of age or older and meet set criteria for participating in the study. You are asked to participate since you meet the criteria. If you accept participation in the study, you will either receive usual care or one of the two exercise interventions. One exercise intervention contains strength, balance and walking exercises and the other exercise intervention contains chair-stand exercises and walking. The exercise you receive through your participation in the project is in addition to the usual care. Whether you receive usual care, or one of the two training interventions is determined by the period of time you are hospitalized.

The research principal for the project is Karolinska Institutet. Research principal means the organization responsible for the study. The application has been approved by the Ethics Review Authority, the diary number for the review at the Ethics Review Authority is 2022-03032-01.

How is the study conducted?

Your participation in this research study will mean that at the beginning and end of your hospital stay and approximately three months after discharge, you will be asked to do tests and answer questionnaires. The tests aim to examine walking speed, balance, leg strength, grip strength, arm and calf circumference, as well as ability to perform activities of daily living. The questionnaires aim to ask you how you perceive your health and quality of life in various areas. This examination will take approximately 30 minutes to complete. The examination, which is carried out three months after discharge, can be done in your home or at the hospital as agreed. Other information such as age, sex, your medical condition, length of hospital stay, and place of discharge will be collected from your medical record. If you choose to participate, you also agree to us collecting information about your visits and diagnoses from the healthcare system up to one year after you have been discharged from the hospital.

The individualized exercise program involves exercising for 40 minutes divided into two sessions per day. Exercises that can be included are strength, balance, and walking. The second exercise program involves exercising for 20–30 minutes spread over four sessions per day. This exercise includes getting up from a chair and walking. You will receive a simple self-training program with these exercises that you continue with at home for six weeks. You register the exercise in a logbook with a cross. All exercise at the hospital is adapted to your ability. The training is led and documented by certified personnel.

Possible consequences and risks of participating in the study

Tests of balance can challenge your balance. In order not to risk losing your balance and fall, there will be a person close to you who will be ready to assist you.

For peer review only

The "Preventing functional decline in acutely hospitalized older patients (PREV_FUNC)" study

What will happen to my data?
The information collected about you in the study will be protected by confidentiality according to the Swedish Publicity and Confidentiality Act, which means that no unauthorized persons may access the information. The collected material will be coded, and the data and code key will be kept separately locked according to standard practice. This means that no one except the responsible researchers can identify you as a person if necessary. Everyone who works on the study is bound by confidentiality. All results from the study will be presented at group level. If during the examination we discover something that needs to be followed up, we can arrange contact with the relevant care facility.
Responsible for your personal data is Karolinska Institutet as the research principal. According to the EU's data protection regulation, you have the right to access the information about you, that is handled in the study, free of charge, and if necessary to have any errors corrected. You can also request that information about you be deleted and that the processing of your personal data be restricted. If you want to access the data, please contact Anna-Karin Welmer, Karolinska Institutet, Department of Physiotherapy, Alfred Nobels allé 23, 141 83 Huddinge, Sweden, telephone number: +46 8 524 888 04. The code key will be destroyed after 10 years. Thereafter, it is not possible to hand out any register extract. The information you provide is protected according to chapter 24. paragraph 8, of the Swedish Publicity and Privacy Act (2009:400) and the EU's data protection regulation, GDPR. The Data Protection Officer can be reached at dataskyddsombud@ki.se. If you are dissatisfied with the way your personal data is processed, you have the right to file a complaint with the Swedish Authority for Privacy Protection (IMY), which is the supervisory authority.

How do I get information about the results of the study?
The study results will be reported at group level and published in scientific journals, popular science literature and at conferences nationally and internationally. Your identity will not be traceable in these reports. If you as a research participant want to get your results out, please contact the researcher responsible for the project.

Insurance and compensation
Compensation is not eligible. Patient insurance covers your participation in the study.

Participation is voluntary
Your participation is voluntary, and you can choose to cancel your participation at any time. If you choose not to participate or wish to cancel your participation, you do not need to state why, and it will not affect your future care or treatment. If you wish to cancel your participation, please contact the responsible researchers (see below).

Responsible for the study
For further information, please contact:

Anna-Karin Welmer<br>Senior Lecturer, Associate Professor, Physiotherapist<br>anna-karin.welmer@ki.se<br>+46 8 524 888 04	Anne-Marie Boström<br>Senior Lecturer, Associate Professor, RN<br>anne-marie.bostrom@ki.se<br>+46 760 519513	Linda Sandberg<br>PhD, Occupational Therapist<br>linda.m.sandberg@ki.se

For peer review only

The "Preventing functional decline in acutely hospitalized older patients (PREV_FUNC)" study

Consent to participate in the study

I have received oral and written information about the study and have had the opportunity to ask questions. I get to keep the written information.

☐ I agree to participate in the study *Preventing functional decline in acutely hospitalized older patients (PREV_FUNC)*

☐ I agree to data about me being collected and processed in the manner described in the research participant information

Place and date	Signature
	Name claification

For peer review only

SPIRIT

STANDARD PROTOCOL ITEMS: RECOMMENDATIONS FOR INTERVENTIONAL TRIALS

SPIRIT 2013 Checklist: Recommended items to address in a clinical trial protocol and related documents*

Section/item	Item No	Description	Addressed on page number
**Administrative information**			
Title	1	Descriptive title identifying the study design, population, interventions, and, if applicable, trial acronym	Page 1, Title page
Trial registration	2a	Trial identifier and registry name. If not yet registered, name of intended registry	Page 3, Abstract
	2b	All items from the World Health Organization Trial Registration Data Set	
Protocol version	3	Date and version identifier	Page 15, Paragraph 1
Funding	4	Sources and types of financial, material, and other support	Page 19, Funding statement
Roles and responsibilities	5a	Names, affiliations, and roles of protocol contributors	Page 1, Title page and page 19, Author contributions
	5b	Name and contact information for the trial sponsor	Page 19, Funding statement
	5c	Role of study sponsor and funders, if any, in study design; collection, management, analysis, and interpretation of data; writing of the report; and the decision to submit the report for publication, including whether they will have ultimate authority over any of these activities	Page 19, Funding statement

For peer review only

	5d	Composition, roles, and responsibilities of the coordinating centre, steering committee, endpoint adjudication committee, data management team, and other individuals or groups overseeing the trial, if applicable (see Item 21a for data monitoring committee)	N/A
**Introduction**			
Background and rationale	6a	Description of research question and justification for undertaking the trial, including summary of relevant studies (published and unpublished) examining benefits and harms for each intervention	Page 5-6, Introduction
	6b	Explanation for choice of comparators	Page 5-6, Introduction, Paragraph 4
Objectives	7	Specific objectives or hypotheses	Page 6, Introduction, last paragraph
Trial design	8	Description of trial design including type of trial (eg, parallel group, crossover, factorial, single group), allocation ratio, and framework (eg, superiority, equivalence, noninferiority, exploratory)	Page 6, Trial design
**Methods: Participants, interventions, and outcomes**			
Study setting	9	Description of study settings (eg, community clinic, academic hospital) and list of countries where data will be collected. Reference to where list of study sites can be obtained	Page 6, Trial design
Eligibility criteria	10	Inclusion and exclusion criteria for participants. If applicable, eligibility criteria for study centres and individuals who will perform the interventions (eg, surgeons, psychotherapists)	Page 7, Inclusion and exclusion criteria, Page 7-8 Interventions

For peer review only - http://bmjopen.bmj.com/site/about/guidelines.xhtml

Interventions	11a	Interventions for each group with sufficient detail to allow replication, including how and when they will be administered	Page 7-8, Interventions, Page 9-10, Table 1
	11b	Criteria for discontinuing or modifying allocated interventions for a given trial participant (eg, drug dose change in response to harms, participant request, or improving/worsening disease)	N/A
	11c	Strategies to improve adherence to intervention protocols, and any procedures for monitoring adherence (eg, drug tablet return, laboratory tests)	Page 8, last paragraph
	11d	Relevant concomitant care and interventions that are permitted or prohibited during the trial	N/A
Outcomes	12	Primary, secondary, and other outcomes, including the specific measurement variable (eg, systolic blood pressure), analysis metric (eg, change from baseline, final value, time to event), method of aggregation (eg, median, proportion), and time point for each outcome. Explanation of the clinical relevance of chosen efficacy and harm outcomes is strongly recommended	Page 11-12, Data collection, Page 13, Table 2
Participant timeline	13	Time schedule of enrolment, interventions (including any run-ins and washouts), assessments, and visits for participants. A schematic diagram is highly recommended (see Figure)	Page 15, Trial status, Figure 1
Sample size	14	Estimated number of participants needed to achieve study objectives and how it was determined, including clinical and statistical assumptions supporting any sample size calculations	Page 14, Data analysis
Recruitment	15	Strategies for achieving adequate participant enrolment to reach target sample size	Page 6, Trial design

Methods: Assignment of interventions (for controlled trials)

Allocation:

Sequence generation	16a	Method of generating the allocation sequence (eg, computer-generated random numbers), and list of any factors for stratification. To reduce predictability of a random sequence, details of any planned restriction (eg, blocking) should be provided in a separate document that is unavailable to those who enrol participants or assign interventions	Page 6-7, Trial design

Allocation concealment mechanism	16b	Mechanism of implementing the allocation sequence (eg, central telephone; sequentially numbered, opaque, sealed envelopes), describing any steps to conceal the sequence until interventions are assigned	Page 6-7, Trial design
Implementation	16c	Who will generate the allocation sequence, who will enrol participants, and who will assign participants to interventions	Page 6-7, Trial design
Blinding (masking)	17a	Who will be blinded after assignment to interventions (eg, trial participants, care providers, outcome assessors, data analysts), and how	Page 8, Data collection
	17b	If blinded, circumstances under which unblinding is permissible, and procedure for revealing a participant's allocated intervention during the trial	N/A
**Methods: Data collection, management, and analysis**			
Data collection methods	18a	Plans for assessment and collection of outcome, baseline, and other trial data, including any related processes to promote data quality (eg, duplicate measurements, training of assessors) and a description of study instruments (eg, questionnaires, laboratory tests) along with their reliability and validity, if known. Reference to where data collection forms can be found, if not in the protocol	Page 11-12, Data collection
	18b	Plans to promote participant retention and complete follow-up, including list of any outcome data to be collected for participants who discontinue or deviate from intervention protocols	Page 11-12, Data collection, Secondary outcomes
Data management	19	Plans for data entry, coding, security, and storage, including any related processes to promote data quality (eg, double data entry; range checks for data values). Reference to where details of data management procedures can be found, if not in the protocol	Page 15, Ethics and dissemination
Statistical methods	20a	Statistical methods for analysing primary and secondary outcomes. Reference to where other details of the statistical analysis plan can be found, if not in the protocol	Page 14, Data analysis
	20b	Methods for any additional analyses (eg, subgroup and adjusted analyses)	Page 14, Data analysis

	20c	Definition of analysis population relating to protocol non-adherence (eg, as randomised analysis), and any statistical methods to handle missing data (eg, multiple imputation)	Page 14, Data analysis
**Methods: Monitoring**			
Data monitoring	21a	Composition of data monitoring committee (DMC); summary of its role and reporting structure; statement of whether it is independent from the sponsor and competing interests; and reference to where further details about its charter can be found, if not in the protocol. Alternatively, an explanation of why a DMC is not needed	N/A
	21b	Description of any interim analyses and stopping guidelines, including who will have access to these interim results and make the final decision to terminate the trial	N/A
Harms	22	Plans for collecting, assessing, reporting, and managing solicited and spontaneously reported adverse events and other unintended effects of trial interventions or trial conduct	Page 8, last paragraph
Auditing	23	Frequency and procedures for auditing trial conduct, if any, and whether the process will be independent from investigators and the sponsor	Page 19, Funding statement
**Ethics and dissemination**			
Research ethics approval	24	Plans for seeking research ethics committee/institutional review board (REC/IRB) approval	Page 11, Ethics and dissemination
Protocol amendments	25	Plans for communicating important protocol modifications (eg, changes to eligibility criteria, outcomes, analyses) to relevant parties (eg, investigators, REC/IRBs, trial participants, trial registries, journals, regulators)	Page 10, Trial status
Consent or assent	26a	Who will obtain informed consent or assent from potential trial participants or authorised surrogates, and how (see Item 32)	Page 15, Ethics and dissemination
	26b	Additional consent provisions for collection and use of participant data and biological specimens in ancillary studies, if applicable	N/A

For peer review only - http://bmjopen.bmj.com/site/about/guidelines.xhtml

Confidentiality	27	How personal information about potential and enrolled participants will be collected, shared, and maintained in order to protect confidentiality before, during, and after the trial	Page 15, Ethics and dissemination
Declaration of interests	28	Financial and other competing interests for principal investigators for the overall trial and each study site	Page 19, Competing interests
Access to data	29	Statement of who will have access to the final trial dataset, and disclosure of contractual agreements that limit such access for investigators	Page 15, Ethics and dissemination
Ancillary and post-trial care	30	Provisions, if any, for ancillary and post-trial care, and for compensation to those who suffer harm from trial participation	N/A
Dissemination policy	31a	Plans for investigators and sponsor to communicate trial results to participants, healthcare professionals, the public, and other relevant groups (eg, via publication, reporting in results databases, or other data sharing arrangements), including any publication restrictions	Page 15, Ethics and dissemination
	31b	Authorship eligibility guidelines and any intended use of professional writers	Page 15, Ethics and dissemination
	31c	Plans, if any, for granting public access to the full protocol, participant-level dataset, and statistical code	N/A
**Appendices**			
Informed consent materials	32	Model consent form and other related documentation given to participants and authorised surrogates	Appendix 1
Biological specimens	33	Plans for collection, laboratory evaluation, and storage of biological specimens for genetic or molecular analysis in the current trial and for future use in ancillary studies, if applicable	N/A

*It is strongly recommended that this checklist be read in conjunction with the SPIRIT 2013 Explanation & Elaboration for important clarification on the items. Amendments to the protocol should be tracked and dated. The SPIRIT checklist is copyrighted by the SPIRIT Group under the Creative Commons "Attribution-NonCommercial-NoDerivs 3.0 Unported" license.

For peer review only - http://bmjopen.bmj.com/site/about/guidelines.xhtml
